# Azygos vein preservation revisited: impact on early outcomes after repair of esophageal atresia/tracheoesophageal fistula in newborns

**DOI:** 10.1007/s13304-023-01684-4

**Published:** 2023-11-09

**Authors:** Mohammad Daboos, Mohamed Abdelmaboud, Mohamed Hussein, Ahmed Salama, Ahmed Elshamy

**Affiliations:** 1 Department of Pediatric Surgery, Al-Azhar University, Al-Houssain University Hospital, Darrasa, Cairo, Egypt; 2 Pediatric Surgery Unit, Department of Surgery, Al-Azhar University, Assuit, Egypt

**Keywords:** Esophageal atresia, Tracheoesophageal fistula, Preservation of the Azygos vein, Division of Azygos vein, Pediatrics

## Abstract

Since the first successful repair of esophageal atresia/tracheoesophageal fistula (EA–TEF) was performed approximately 8 decades ago, surgeons have made considerable technical advances in solving intraoperative surgical challenges and reducing postoperative complications. According to some surgeons, preserving the Azygos vein makes this modification attractive. This study aimed to evaluate the benefits of preserving the Azygos vein during surgery for esophageal atresia with tracheoesophageal fistula and to highlight its advantages in reducing anastomotic leak, stricture, and other postoperative outcomes. This prospective comparative series was conducted between April 2020 and April 2023. The study included all newborns with EA–TEF eligible for primary repair. Patients were randomized to either Group A or B. Group A underwent Azygos vein preservation, whereas the remaining patients (Group B) underwent Azygos vein disconnection. Sixty-four patients were included in this study. Thirty-two patients (Group A) underwent Azygos vein preservation during EA–TEF repair, and the remaining thirty-two patients (Group B) underwent Azygos vein ligation and disconnection. Both groups were comparable in terms of demographics, clinical data, and operative findings (*P* > 0.05). Pneumonitis occurred in 4 patients in Group A and 16 patients in Group B. Anastomotic leaks occurred in two (6.2%) patients in Group A and six (18.7%) patients in Group B. There were two deaths in Group A and six deaths in Group B, with a significant difference between the two groups (*P* = 0.0485). Preserving the Azygos vein during esophageal atresia repair reduces the occurrence of postoperative pneumonia, leakage, and stenosis, and decreases postoperative mortality. Therefore, we declare that this modification is a significant and valuable addition to the current surgical procedures.

## Introduction

Esophageal atresia/tracheoesophageal fistula is a rare congenital anomaly that affects 1 in every 10,000 live births, challenging the technical and diagnostic skills of surgeons. Most pediatric surgeons consider treating esophageal atresia as the pinnacle of newborn surgical care [1]. Early postoperative complications included anastomotic leaks, esophageal strictures, recurrence of tracheoesophageal fistula (TEF), pneumonia, and sepsis. Long-term complications associated with this operation include tracheomalacia, gastric reflux, dysphagia, recurrent pneumonia, and cough [2]. It has been over 8 decades since the first successful repair of EA–TEF [1]. Since then, surgeons have made significant technical advances in solving intraoperative surgical challenges, reducing postoperative complications, and enhancing postoperative surgical outcomes. However, the essential concept underlying this approach remains unchanged [3]. Another interesting modification is the preservation of the Azygos vein [4]. However, other studies have revealed no relationship between the preservation of the Azygos vein and the prevalence of anastomotic complications and/or pneumonia [5, 6]. In contrast, several studies have found that neonates with preserved veins after repair have a decreased incidence of early postoperative complications such as anastomotic leaks, strictures, and chest infections [7–9]. In this study, we compared the preservation of the Azygos vein and its disconnection in the surgical repair of EA–TEF with tracheoesophageal fistula. This study aimed to evaluate the impact of vein preservation on early surgical outcomes.

## Patients and methods

This prospective comparative series was conducted at Al-Azhar University Hospitals to assess the technical feasibility and surgical outcomes of EA–TEF repair, with or without Azygos vein preservation, in newborns diagnosed with EA–TEF between April 2020 and April 2023. This study included a series of cases who were eligible for the primary repair of EA–TEF. Patients with long-gap esophageal atresia (> 3 cm) or atresia without TEF were excluded from the study because of the significant impact of long gaps on postoperative complications. Using the sealed, closed envelope method, patients were randomly assigned to one of the two groups. All eligible patients who underwent EA–TEF repair with Azygos vein preservation were included in Group A (32 cases), whereas those who underwent EA–TEF repair with Azygos vein ligation and disconnection were included in Group B (32 cases). This study followed the principles of the Declaration of Helsinki and was approved by the Ethics Committee of our university (Faculty of Medicine, Al-Azhar University). Before the surgery, the patient’s legal representative signed an informed consent form. The study was registered in the Clinical Trial Registry. Numbered as (NCT05957562).

## Surgical techniques

**Fig. 1:**
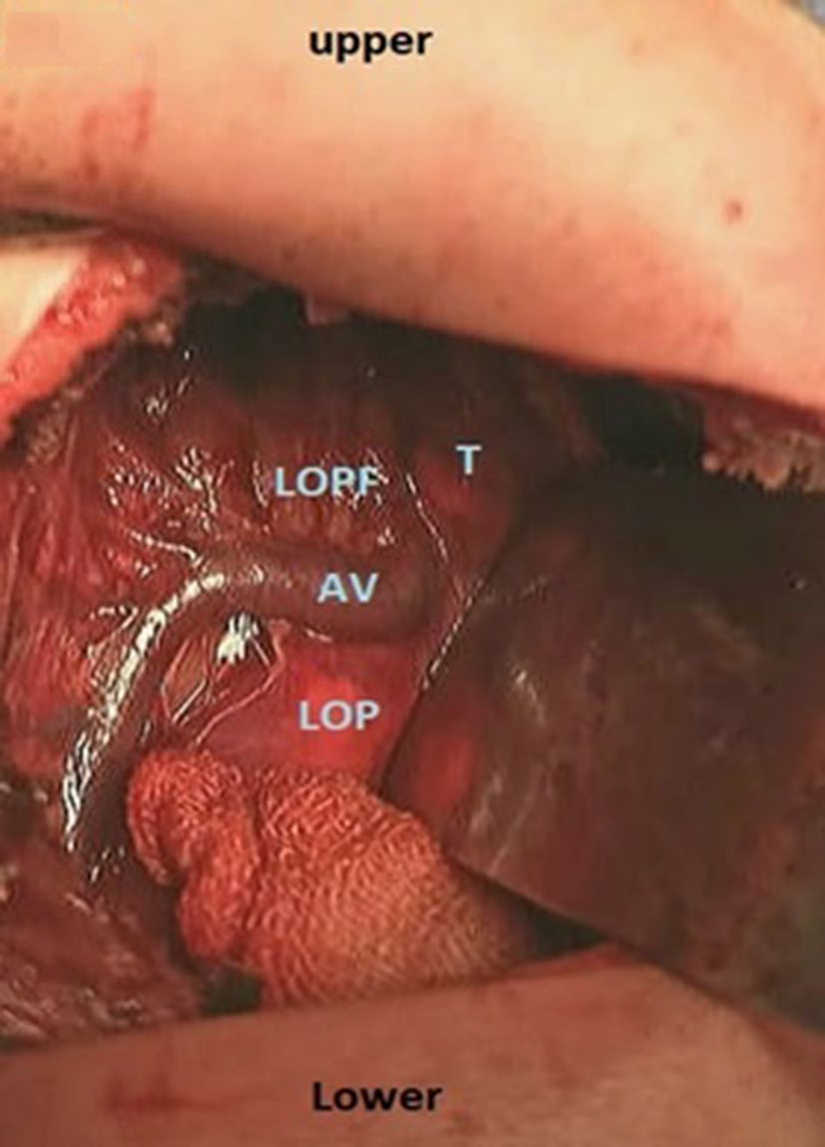
The Azygos vein and the posterior mediastinum were exposed, revealing the lower pouch and fistula. *AV* Azygos vein, *LOP* lower oesophageal pouch, *LOPF* site of lower oesophageal pouch fistula, *T* trachea

The trachea and bronchi were promptly examined using bronchoscopy while the patient was under general anesthesia. The esophageal fistula was mostly located 5–7 mm above the carina. An endotracheal tube was inserted beyond the TEF to avoid the insufflation of gas into the stomach. The infant was then placed on his left side and supported with jelly bags. An incision was made 1 cm below the inferior angle of the right scapula running posteriorly from the mid-axillary line to the paravertebral line. The scapula was lifted after the muscle division. The thorax was accessed via the fourth intercostal space. The pleura was gently detached from the chest wall by gently introducing a gauze swab into the extrapleural space. A rib spreader was used to separate the ribs gently. The pleura was further dissected posteriorly using wet pledgets. Subsequently, the lower pouch and Azygos veins were observed [10] (Fig. 1).

**Fig. 2:**
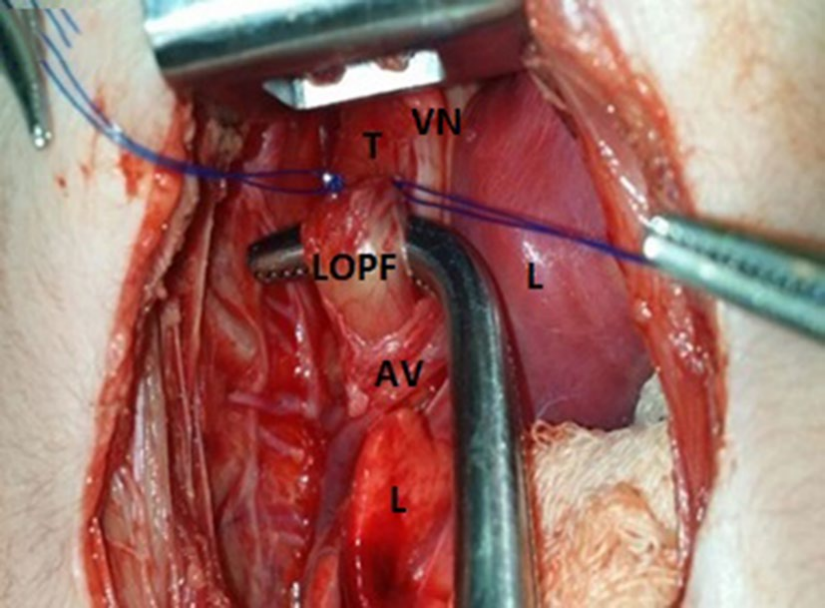
Exposure of the posterior wall of the trachea revealed the tracheoesophageal fistula which was elevated using right angel forceps. *AV* Azygos vein, *LOPF* lower oesophageal pouch fistula, *L* lung, *T* trachea, *VN* vagus nerve

**Fig. 3:**
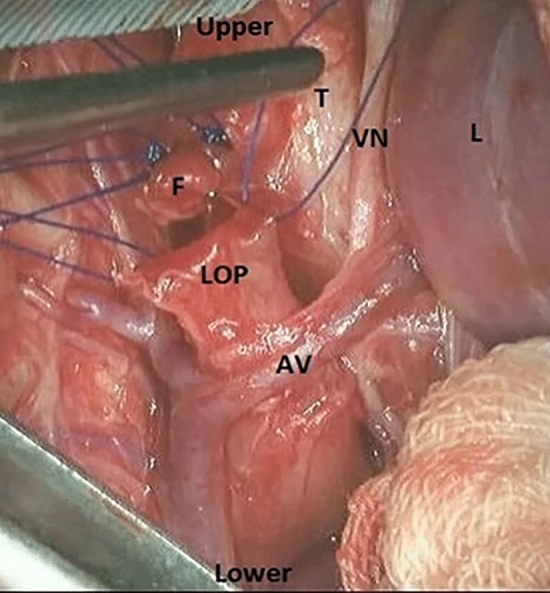
The fistula was divided and closed using a non-absorbable monofilament 6/0 suture. *AV* Azygos vein, *F* fistula, *LOP* lower oesophageal pouch, *L* lung, *T* trachea, *VN* vagus nerve

*In Group A,* we bluntly detached the tissue beneath the trachea without cutting the Azygos vein, as described by Rashid et al. [6]. The lower esophagus was dissected circumferentially until immediately distal to the fistula. The right-angle forceps was positioned below it (Fig. 2). Non-absorbable monofilament 6/0 sutures were used to close the fistula after splitting (Fig. 3).

*In Group B*, the Azygos vein was divided and ligated following exposure. The lower esophagus was dissected circumferentially, and the fistula was isolated and closed using non-absorbable monofilament 6/0 sutures.

**Fig. 4:**
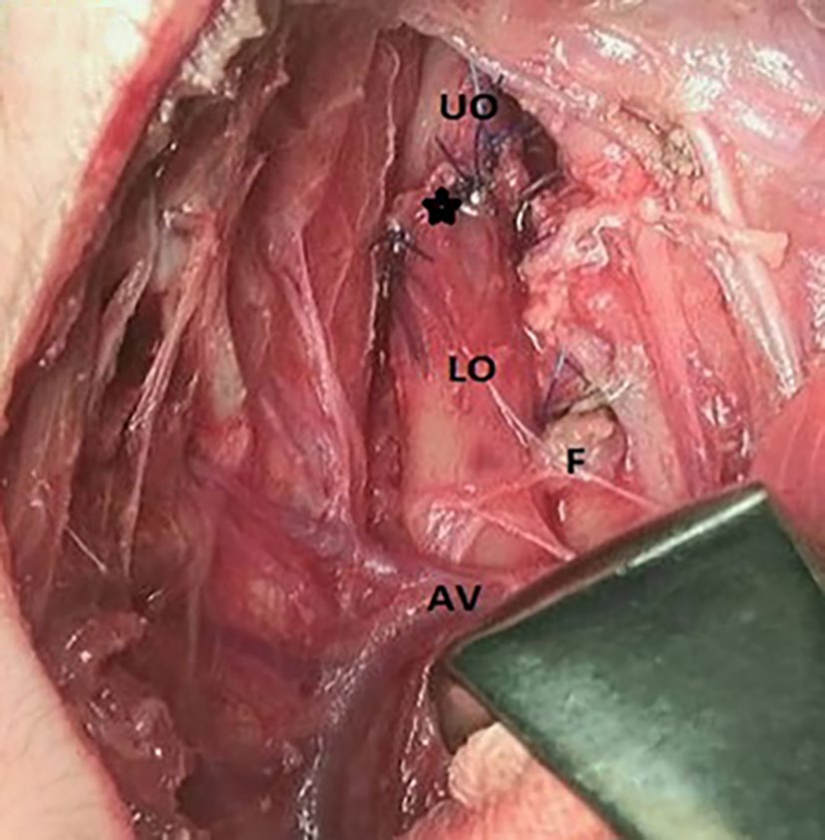
The anastomosis was completed medial to the Azygos vein. *AV* Azygos vein, *F* fistula, *LO* lower oesophagus, *UO* upper oesophagus, black asterisk point to anastomosis

*In both groups*, after instilling warm saline solution into the chest, fistula closure was assessed for air leaks by observing bubbles during forced respiration. The upper pouch was identified. Traction and dissection were performed to enable simple anastomosis. Interrupted absorbable 5/0 sutures were used to create an end-to-end anastomosis medial to the Azygos vein in Group A. The distal end of a 5F silicone feeding tube was inserted into the stomach after anastomosis was established (Fig. 4).

**Fig. 5:**
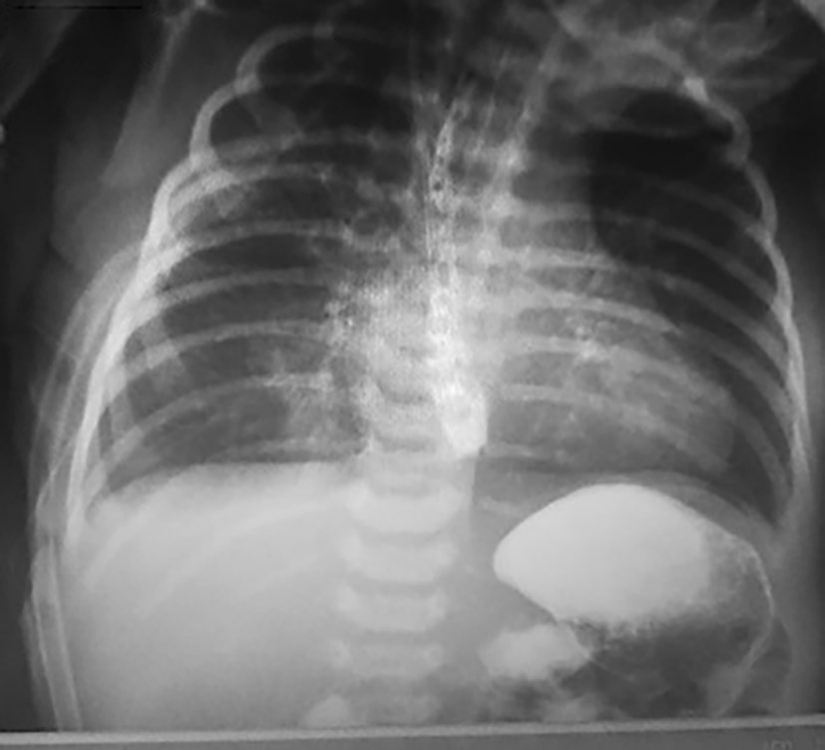
Water-soluble contrast esophagography on the 6th postoperative day showing no signs of leakage or stenosis

All patients began receiving feeding through a trans-anastomotic tube 72 h after surgery. After performing water-soluble contrast esophagography (Fig. 5), oral feeding was initiated on the 6th postoperative day. Patients were followed up at 1, 3, 6, and 12 months after surgery.

The groups were compared in terms of gestational age, sex, birth weight, associated anomalies, Spitz classification, gap between the pouches after mobilization, operative time, and early postoperative complications, such as pneumonia, anastomotic leaks, anastomotic stenosis, and mortality.

## Calculated sample size = (64)

Population size 76, population proportion 50%, confidence level 95%, margin of error 5%.

### Method of randomization and patient selection

The sample size was determined before beginning the study. We then prepared 64 nontransparent envelopes, with each envelope containing a card. Out of the 64 envelopes, 32 contained cards signed for “esophageal atresia repair with Azygos vein preservation” and the other 32 envelopes contained cards signed for “esophageal atresia repair with Azygos vein disconnection.” All envelopes were closed and mixed. Each patient came one by one, and each patient's parent blindly chose one of the closed envelopes. The surgical committee opened the envelopes to determine the chosen technique. This method is called the permuted block design, and it is used to ensure that both groups in the current study have equal and balanced numbers.

### Statistical analysis

Statistical Package for Social Science (SPSS, version 23) was used to analyze the data. Parametric data are presented as mean and standard deviation. Numbers and percentages were used to represent qualitative characteristics. The Kolmogorov–Smirnov and Shapiro–Wilk tests were used to assess the normality of the data distribution. Using qualitative data, the Chi-squared test was used to compare the groups. The comparison between two groups with quantitative data and parametric distributions was done using an independent *t* test.

## Results

The present study included 64 patients who were divided into 2 groups. Azygos vein preservation was performed in 32 patients (Group A). The remaining 32 patients (Group B) had their Azygos veins ligated and disconnected. Both groups had comparable sex ratios. Group A included 20 males and 12 females, while Group B included 18 males and 14 females. The mean gestational age was comparable between the two groups. It was 36 ± 1.4 weeks in Group A and 37 ± 1.1 weeks in Group B. In Group A, 50% (16/32) of patients had associated abnormalities, while in Group B, 37.5% (12/32) of patients had associated abnormalities. The mean birth weight in Group A was 2.4 ± 0.2 kg, while it was 2.35 ± 0.25 kg in Group B. Group A comprised 2 cases of type 2 and 30 cases of type 1, according to the Spitz classifications, while Group B had comparable findings (Table 1). There were no significant differences between the two groups in terms of Spitz risk classification criteria. There were no statistically significant differences in the demographic data or patient characteristics between the groups.

**Table 1:** Demographic preoperative data

Demographic data	Group A (*n* = 32)	Group B (*n* = 32)	Test value	*p* value
Sex				
Male	20 (62.5%)	18 (56.25%)	0.130*	0.718
Female	12 (37.5%)	14 (43.75%)		
Gestational age/week (mean)	36** ± **1.4	37** ± **1.1	1.247•	0.052
Weight (kg)	2.4** ± **0.2	2.35** ± **0.25	0.625•	0.536
Associated anomalies				
Cardiac	8 (25.0%)	10 (31.3%)	0.155*	0.693
Gastrointestinal	2 (6.3%)	0 (0.0%)	1.032*	0.309
Vertebral	2 (6.3%)	0 (0.0%)	1.032*	0.309
Musculoskeletal	0 (0.0%)	0 (0.0%)	0.000*	1.000
Genitourinary	4 (12.5%)	2 (6.5%)	0.368*	0.544
Total	16 (50.0%)	12 (37.5%)	0.508*	0.476
Spitz risk classifications				
1-B. W > 1.500 g otherwise healthy	30 (93.8%)	30 (93.8%)	0.000*	1.000
2-B. W < 1.500 g or major cardiac anomaly	2 (6.3%)	2 (6.3%)		
3-B. W < 1.500 g with major cardiac anomaly	0 (0.0%)	0 (0.0%)		

•Independent sample t test for mean ± SD

^*^Χ^2^ test for percentages

*p* value > 0.05 is insignificant; *p* value < 0.05 is significant; *p* value < 0.01 is highly significant

The gap distance between the two esophageal pouches was quite similar in both groups after mobilization. The mean distance in Group A was 1.3 ± 0.5 cm, whereas in Group B, it was 1.4 ± 0.3 cm. There were no significant differences in the operating times and the length of hospital stay between Groups A and B. The mean operative times were 65 ± 11 and 62 ± 12 min in Groups A and B, respectively. The mean length of hospital stay was 12 ± 2.1 days in Group A and 13 ± 2.4 days in Group B (Table 2).

**Table 2:** Operative data (esophageal gap distance, operative time) and length of hospital stay

Parameters	Group A (*n* = 32)	Group B (*n* = 32)	Test value	*p* value
Gap distance/cm	1.3 ± 0.5	1.4 ± 0.3	0.686•	0.498
Operative time/minutes	65 ± 11	62 ± 12	0.737•	0.467
Mean post-operative hospital stay/days	12 ± 2.1	13 ± 2.4	0.509•	0.751

•Independent sample *t* test for mean ± SD

*p* value > 0.05 is insignificant; *p* value < 0.05 is significant; *p* value < 0.01 is highly significant

In the context of postoperative complications, 16/32 patients (50%) in Group B experienced pneumonitis in the postoperative period compared to 4/32 (12.5%) in Group A. There were statistically significant differences between the two groups. Six cases (18.7%) of anastomotic leakage occurred in Group B, compared with 2/32 (6.2%) in Group A. Most cases were managed conservatively in both groups, except for two cases of major leakage in Group B that necessitated surgical intervention and repair. There were six cases (18.7%) of esophageal strictures in Group B, compared to two (6.2%) in Group A (Table 3). Postoperative anastomotic leakage and stricture were significantly different between the two groups. Except for one case in Group B, which had a severe esophageal stricture that did not respond to dilation and required thoracotomy for redo anastomosis, the majority of esophageal strictures were effectively treated with dilation.

**Table 3:** Postoperative complications

	Group A (*n* = 32)	Group B (*n* = 32)	Test value*	*p* value
Pneumonitis	4 (12.5%)	16 (50.0%)	5.236	0.022
Anastomotic leaks				
Minor	2 (6.3%)	4 (12.5%)	0.368	0.544
Major	0 (0.0%)	2 (6.3%)	1.032	0.309
Total leaks	2 (6.3%)	6 (18.8%)	2.143	0.0485
Anastomotic stricture	2 (6.3%)	6 (18.8%)	2.143	0.0485
Mortality	2 (6.25%)	6 (18.8%)	2.143	0.0485

^*^Χ^2^ test for percentages

*p* value > 0.05 is insignificant; *p* value < 0.05 is significant; *p* value < 0.01 is highly significant

The mortality rates in the two groups were significantly different. Two patients in Group A died of acute pneumonia, while in Group B, two patients died due to septicemia following a major anastomotic leak, two due to significant cardiac abnormalities and severe chest infection, and two due to a significant chest infection and subsequent septicemia (Table 3). However, multivariate analysis showed that gestational age and major cardiac anomalies were the main risk factors for mortality in both groups (Table 4).

**Table 4:** Multivariate analysis

	Multivariate analysis			
	*p*	OR (LL–UL 95% CI)	*p*	^#^AOR (LL–UL 95%CI)
Leakage	0.0485	0.289 (0.054–1.557)	0.0485	0.289 (0.053–1.568)
Stricture	0.0485	0.289 (0.054–1.557)	0.0485	0.289 (0.053–1.568)
Pneumonia	0.002	0.143 (0.041–0.502)	0.002	0.082 (0.017–0.410)
Mortality	0.0485	0.289 (0.054–1.557)	0.0485	0.289 (0.053–1.568)

*OR* odd’s ratio, ^#^*AOR* adjust odd’s ratio by gestational age and major cardiac anomalies, *p* value for univariate regression analysis; *p* value > 0.05 is insignificant; *p* value < 0.05 is significant

## Discussion

Since Cameron Haight described the first successful primary repair of EA–TEF by performing esophageal anastomosis and fistula division in 1943, few modifications have been made to this surgical procedure [11, 12]. The preservation of the Azygos vein is a modification that has received significant attention and yielded some interesting results. The cavo-caval bypass includes the Azygos vein, an important unpaired vein formed by the confluence of the right ascending lumbar veins with the right subcostal veins. In addition to obtaining blood from the pericardium, mediastinum, bronchus, and esophagus, this venous system also receives lymphatic vessels directly from the thoracic duct. The Azygos vein, therefore, maintains mediastinal venous drainage, minimizing postoperative esophageal congestion and edema [7, 9, 13, 14].

In their meta-analysis on the influence of Azygos vein preservation, Kainth et al. recommended conducting a randomized controlled trial with a large sample size using a standardized operating technique for the surgical repair of esophageal atresia/tracheoesophageal fistula. Consequently, the two operating approaches employed in our study, which were standardized by the same surgical team, were used to compare the surgical outcomes of the two groups of TEF patients and to evaluate the effects of preserving the Azygos vein on surgical outcomes [15].

In this study, we performed a tailored esophageal anastomosis medial to the Azygos arch. Although esophageal anastomosis to the preserved Azygos arch can be performed either medially or laterally, it is commonly performed laterally owing to its simplicity. Rashid et al. [6] revealed in their study that the anastomosis medial to the preserved Azygos arch was not technically demanding, and we agree with them.

Bronchial veins can flow into the Azygos arch [6, 11]. Thus, ligation of the Azygos vein induces bronchial wall edema, luminal obstruction, and pneumonitis [13, 14]. Furthermore, Soyer et al. [8] and Upadhyaya et al. [13] reported that pneumonitis and lung congestion were more common in individuals undergoing Azygos vein ligation. In addition, the current study revealed that 50% (16/32) of the patients in the Azygos vein ligation group experienced postoperative pneumonitis. This finding is consistent with those reported by Soyer et al. [8] and Upadhyaya et al. [13].

Sharma et al. [7] reported that anastomotic leaks occurred in 20% (10/50) of cases in the group that underwent Azygos vein ligation with TEF repair, compared to 6% (3/46) in the Azygos vein preservation group. In the present study, there were six cases (18.7%) of leakage in the Azygos vein ligation group, including two major leaks and four minor leaks. However, Sharma et al. [7] reported that the ligation group had a higher incidence of major leaks, which could be attributed to the larger sample size in their study. Anastomotic leakage is influenced by several factors, including the amount of exudate at the site of anastomosis, degree of tissue edema, adequacy of blood supply, and technical errors [9]. According to Cui et al. [9], anastomotic leakage occurred in 5.9% of patients with preserved Azygos veins and in 19.4% of patients with divided veins in their series. The results of the current study showed that the rate of anastomotic leakage was 6.2% in the preservation group and 18.7% in the division group, which is comparable to the findings of Cui et al. [9].

A meta-analysis conducted by Wang et al. [16] provided compelling evidence regarding the safety and benefits of preserving the Azygos vein in patients with EA–TEF. They demonstrated that preserving the Azygos vein can reduce the incidence of anastomotic leakage compared to ligating it. In addition, they recommended evaluating other aspects, such as postoperative esophageal strictures, in accordance with our study [16].

Cui et al. [9] reported higher rates of esophageal stricture in groups that underwent Azygos vein ligation, which is consistent with the findings of the current study. Their findings showed that anastomotic strictures were related to inflammation and scar hyperplasia caused by anastomotic leakage. As a result, anastomotic leakage was less prevalent after Azygos vein preservation and anastomotic stenosis was also less common [9, 17].

The risk of death was not only related to hemodynamic stability caused by Azygos vein ligation but also to severe anastomotic leak and pneumonia, as described by Upadhyaya et al. [13]. Sharma et al. [7] reported six deaths attributed to a significant leak in the Azygos vein division group. According to Rashid et al. [6], severe pneumonitis, septicemia, and concurrent abnormalities are associated with an increased mortality rate in patients who undergo division of the Azygos vein. All previous findings were confirmed by the results of the present study.

It makes sense to advise against dividing the Azygos vein during EA–TEF repair, as it is crucial for venous drainage. This modification of the original treatment is recommended because of the numerous benefits of preserving this vein, as indicated in the present study. Furthermore, if the theory linking multiple sclerosis to failure of the cerebrospinal fluid venous drainage system is true, it may underscore the crucial role of preserving the Azygos vein. [18–20].

## Study limitations

The limitation of this study is that it was a single-center study with a series of TEF cases; therefore, future research from multiple centers with a larger number of cases is mandatory to accurately assess the effectiveness of this technique. In addition, the follow-up period in this study was brief, and a long-term follow-up period is required. The study had no specific or innovative trials, making the methodology, interventions, and results easily generalizable and applicable.

## Conclusion

Preserving the Azygos vein during esophageal atresia repair reduces the occurrence of postoperative pneumonia, leakage, and stenosis and decreases postoperative mortality. Therefore, we declare that this modification is a significant and valuable addition to the current surgical procedures. However, we recommend conducting studies at multiple centers with a larger sample size.

## Data Availability

The datasets used and/or analyzed during the current study are available from the corresponding author but could not be sent owing to the medicolegal aspect of the hospital policy.
